# A Network Pharmacology Study of Chinese Medicine QiShenYiQi to Reveal Its Underlying Multi-Compound, Multi-Target, Multi-Pathway Mode of Action

**DOI:** 10.1371/journal.pone.0095004

**Published:** 2014-05-09

**Authors:** Xiang Li, Leihong Wu, Wei Liu, Yecheng Jin, Qian Chen, Linli Wang, Xiaohui Fan, Zheng Li, Yiyu Cheng

**Affiliations:** 1 Pharmaceutical Informatics Institute, College of Pharmaceutical Sciences, Zhejiang University, Hangzhou, China; 2 State Key Laboratory of Modern Chinese Medicine, Tianjin University of Traditional Chinese Medicine, Tianjin, China; Michigan State University, United States of America

## Abstract

Chinese medicine is a complex system guided by traditional Chinese medicine (TCM) theories, which has proven to be especially effective in treating chronic and complex diseases. However, the underlying modes of action (MOA) are not always systematically investigated. Herein, a systematic study was designed to elucidate the multi-compound, multi-target and multi-pathway MOA of a Chinese medicine, QiShenYiQi (QSYQ), on myocardial infarction. QSYQ is composed of *Astragalus membranaceus* (Huangqi), *Salvia miltiorrhiza* (Danshen), *Panax notoginseng* (Sanqi), and *Dalbergia odorifera* (Jiangxiang). Male Sprague Dawley rat model of myocardial infarction were administered QSYQ intragastrically for 7 days while the control group was not treated. The differentially expressed genes (DEGs) were identified from myocardial infarction rat model treated with QSYQ, followed by constructing a cardiovascular disease (CVD)-related multilevel compound-target-pathway network connecting main compounds to those DEGs supported by literature evidences and the pathways that are functionally enriched in ArrayTrack. 55 potential targets of QSYQ were identified, of which 14 were confirmed in CVD-related literatures with experimental supporting evidences. Furthermore, three sesquiterpene components of QSYQ, Trans-nerolidol, (3S,6S,7R)-3,7,11-trimethyl-3,6-epoxy-1,10-dodecadien-7-ol and (3S,6R,7R)-3,7,11-trimethyl-3,6-epoxy-1,10-dodecadien-7-ol from *Dalbergia odorifera* T. Chen, were validated experimentally in this study. Their anti-inflammatory effects and potential targets including extracellular signal-regulated kinase-1/2, peroxisome proliferator-activated receptor-gamma and heme oxygenase-1 were identified. Finally, through a three-level compound-target-pathway network with experimental analysis, our study depicts a complex MOA of QSYQ on myocardial infarction.

## Introduction

Chinese medicine, an important branch of the healthcare system in China, has gradually gained popularity both at home and abroad [1]. Chinese medicine, a complex system with guidance from traditional Chinese medicine (TCM) theories, has proven to be especially effective to treat chronic and complex diseases. However, the underlying mechanisms of action (MOA) are rarely investigated systematically.

One of the consensus is that TCM produces its efficacy in a holistic way [2], taking the advantage of multi-target therapy being more effective in combating polygenic diseases than mono-therapies [3]. With the advancements in understanding of pathobiology of human diseases, people find that most diseases are not simply caused by one single factor [4]. It is especially true for the complex multifactorial chronic diseases such as cardiovascular disease (CVD), cancer and diabetes [5], [6]. The TCM treatments can be visualized as a complexity against complexity paradigm between multi-target therapy such as TCM and the complex biological networks of human diseases. The development of analytical tools such as systems biology [7], network biology [8] and network pharmacology [9], [10] provide an opportunity to unravel the complex and holistic mechanisms of TCM in treating complex diseases. In today's post-genomic era, it has become much easier to obtain huge amounts of data related to the effect of drugs on transcriptome of target tissues. Data analysis becomes critical to identify the MOA of drugs. Recently, network models and network analysis of genomic data has been proven useful to translate the microarray information and identify potential targets [11], [12] and enriched pathways involved in the MOA [13]–[15]. Tools such as Cytoscape have often been used to construct and analyze networks [16].

QiShenYiQi (QSYQ) dropping pills are a Chinese medicine prescription approved by the State Food and Drug Administration (SFDA) of China. QSYQ is widely and effectively used in China to treat CVD, including myocardial infarction, angina, myocarditis, myocardial fibrosis and heart failure [17]–[19]. QSYQ is composed of *Astragalus membranaceus* (Huangqi), *Salvia miltiorrhiza* (Danshen), *Panax notoginseng* (Sanqi), and *Dalbergia odorifera* (Jiangxiang). Twelve compounds including astragaloside IV (Ast), calycosin (Cal) and formononetin (For) from Huangqi; danshensu (DSS), protocatechuic aldehyde (PCA) and rosmarinic acid (RSA) from Danshen; ginsenoside Rg1 (Rg1), ginsenoside Rb1 (Rb1) and notoginsenoside R1 (R1) from Sanqi; trans-nerolidol (ENL), (3S,6S,7R)-3,7,11-trimethyl-3,6-epoxy-1,10-dodecadien-7-ol (SDL) and (3S,6R,7R)-3,7,11-trimethyl-3,6-epoxy-1,10-dodecadien-7-ol (RDL) from Jiangxiang are main components of QSYQ, and they were absorbed into blood and distributed into tissues after oral administration [20]. SDL and RDL were identified in Jiangxiang in our previous study [21]. However, the potential targets and the underlying molecular mechanisms of actions of these 12 compounds remain to be systematically elucidated.

In this study, the differentially expressed genes (DEGs) were identified from myocardial infarction (MI) rat model treated with QSYQ, followed by constructing a CVD related compound-target-pathway network linking main compounds to those DEGs supported with literature evidences and the pathways that are functionally enriched in ArrayTrack. The associations of DEGs with CVD were evaluated based on information in database CHD@ZJU of its version 1.0 (http://tcm.zju.edu.cn/chd/) developed by our group [22]. We also manually collected the information of targets reported for 9 compounds (i.e. Ast, Cal, For, DSS, PCA, RSA, Rg1, Rb1 and R1) from literatures in PubMed. The judging criteria of a gene being a target is that a CVD related DEG can be found directly influenced by a compound of QSYQ in literature. Furthermore, as there were no reports on the targets of ENL, SDL and RDL, the potential targets of these three sesquiterpenes were proposed based upon gene expression data and further experimentally validated in this study. Finally, we constructed the compound-target-pathway network on anti-MI of QSYQ to decipher the underlying multi-compound, multi-target and multi-pathway mechanism.

## Materials and Methods

### Cell Lines and Reagents

**Figure 1:**
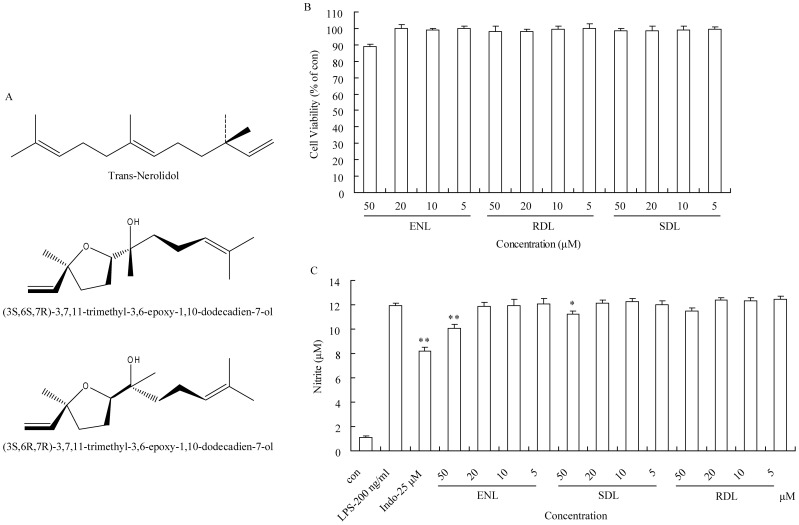
Effects of three sesquiterpene compounds from volatile oil of *Dalbergia odorifera* T. Chen on NO production in LPS-stimulated RAW264.7 macrophages. (A) Structure of ENL, SDL and RDL isolated from *Dalbergia odorifera* T. Chen. (B) Effects of ENL, SDL and RDL on RAW264.7 macrophages viability. (C) Effects of ENL, SDL and RDL on NO production in LPS-stimulated RAW264.7. Indo stands for indomethacin and was used as positive control. Each group was compared with model, and * stands for *p*<0.05, ** stands for *p*<0.01. Each assay was conducted in triplicate and repeated three times. ENL is trans-nerolidol; RDL is (3S,6R,7R)-3,7,11-trimethyl-3,6-epoxy-1,10-dodecadien-7-ol; SDL is (3S, 6S, 7R)-3,7,11-trimethyl-3,6-epoxy-1,10-dodecadien-7-ol.

RAW264.7 cells were purchased from the cell bank of Chinese academy of sciences (Shanghai, China). Trans-nerolidol (ENL) was obtained from Sigma-Aldrich (Cat No. 18143, Sigma, Germany). (3S,6S,7R)-3,7,11-trimethyl-3,6-epoxy-1,10-dodecadien-7-ol (SDL) and (3S,6R,7R)-3,7,11-trimethyl-3,6-epoxy-1,10-dodecadien-7-ol (RDL) was extracted from volatile oil of *Dalbergia odorifera* T. Chen [21] (**Fig. 1A**). Lipopolysaccharide (LPS), U0126 monoethanolate (U0126), T0070907, DMSO, and Thiazolyl blue tetrazolium bromide (MTT) were obtained from Sigma. Pioglitazone hydrochloride (Pio) was purchased from National Institutes for Food and Drug Control (Beijing, China). Nitric oxide assay kit was purchased from Beyotime Institute of Biotechnology (Cat No. S0021, Haimen, Jiangsu, China). Primary antibodies, including anti-ERK1+ERK2 antibody (Cat No. ab17942) for ERK1/2, anti-ERK1+ERK2 (phospho T185+T202+Y204+Y187) antibody (Cat No. ab4819) for pERK1/2, anti-PPAR gamma antibody (Cat No. ab19481) for PPARγ, and anti-Heme Oxygenase 1 antibody [HO-1-1] (Cat No. ab13248) for HO-1, were purchased from Abcam (Hongkong, China).

### Preparation of QiShenYiQi Extracts

QSYQ was prepared from four herbs, *Radix Astragalus membranaceus* (Chinse name Huangqi), *Radix Salvia miltiorrrhiza* (Chinse name Danshen), *Panax notoginseng* (Chinse name Sanqi), and *Dalbergia odorifera* T. Chen (Chinse name Jiangxiang). Briefly, crude drug Huangqi were crushed, for Huangqi, we added water and extracted with refluxing twice, then the extract were filtrated on gauzes. Filtrate was concentrated on a rotary evaporator. In the alcohol sedimentation step, we slowly added 95% ethanol to the filtrate to make alcohol concentration to about 70%. Cooled the solution at room temperature and the supernatant was filtered through cotton gauzes. After recycling the alcohol, the residue was concentrated again to form Huangqi extract. As for crude drug Danshen and Sanqi, they were extracted together with the same procedure as that of Huangqi. For Jiangxiang, we boiled crushed crude drug Jiangxiang with refluxing, and essential oil was extracted in an extraction apparatus. Roots of Sanqi, the trunk and dry heartwood of root from Jiangxiang have been used.

### Animal Study and Gene Expression Data Analysis

Male Sprague-Dawley rats (∼250 g) were obtained from Zhejiang Experimental Animal Center. The Animal Ethic Review Committees of Zhejiang University approved all experimental procedures.

The rats were maintained in air-conditioned rooms (temperature, 22–26°C; humidity, 40%–70%) with a 12-h light-dark cycle, and were acclimatized to the setting. To provide comfortable conditions for rats, each cage contained 5 rats, and the bedding material sawdust was renewed every 2 days. Rats were free to get fresh foods and water, and the water was renewed everyday. All surgery was performed under 10% chloral hydrate anesthesia (300 mg/kg), and all efforts were made to minimize suffering. Rat myocardial infarction was made by occlusion of left anterior descending coronary artery (LAD) according to Yamaguchi [23], [24]. Rats post-operation were laid flat on an electric blanket to help maintain the body temperature of rats. And for those rats which were really weak post-operation, we helped them to recover heart beating with chest cardiac massage and recover breath by connecting the animals with rodent ventilator. For the LAD ligation operation, the mortality was about 40%. Six surviving rats after LAD ligation were randomly divided into myocardial infarction group (MI) and QSYQ-treated group. Three sham-operated rats were treated in the same ways as the rats in MI group but without LAD ligation, which were served as control group (Control). In clinical, QSYQ pills are administered orally administrated to patients with unstable angina and heart failure to prevent acute coronary syndrome. In accordance with clinical recommendations, QSYQ was given intragastrically (i.g.) at a dose of 105.6 mg/kg once a day. Rats in control, and MI groups were administered with saline. Rats were sacrificed after 7 days of i.g. administration under 10% chloral hydrate anesthesia (300 mg/kg). Rats were fixed on a flat plate, and blood samples were collected by abdominal aortic method, then the chest was carefully dissected and the heart was cut quickly. Three tissue samples on the border between infarct and non-infarct area were dissected from left ventricles of each group. The tissue samples were stored at −80°C refrigerator.

Total RNA was extracted using TRIzol Reagent (Invitrogen) and purified using RNeasy Mini kit (QIAGEN), following manufacturers' protocols. RNA quality was evaluated using an Agilent 2100 Bioanalyzer and electrophoresis in 2% (w/v) agarose gels. Only RNA with RNA integrity number (RIN) greater than 7.0 and 28S/18S ratio greater than 0.7 was used for microarray analyses. Whole genome microarray analysis was performed using Affymetrix rat Genome 230 2.0 array. Briefly, total RNA were amplified, labeled, and purified using GeneChip 3′IVT Express Kit (Affymetrix) according to the manufacturer's instructions to obtain biotin labeled cRNA. Array hybridization and wash were performed using GeneChip Hybridization, Wash and Stain Kit in Hybridization Oven 645 (Affymetrix) and Fluidics Station 450 (Affymetrix) following the manufacturer's instructions. Slides were scanned by GeneChip Scanner 3000 and Command Console Software 3.1 with default settings. Spike-in control transcripts were monitored to verify hybridization integrity. The dataset as CEL files was deposited to GEO and the access number is GSE54134. Significant genes were identified through an algorithm developed in-house[25].

As mentioned above, we validated the CVD-related DEGs by manually checking published literatures in PubMed (by April 10^th^, 2013). The keywords used to search papers in PubMed were: “Astragaloside IV”, “Calycosin”, “Formononetin”, “Danshensu”, “Salvianic Acid A”, “Tanshinol”, “Protocatechuic Aldehyde”, “Protocatechualdehyde”, “Rosmarinic Acid”, “Ginsenoside Rg1”, “Ginsenoside Rb1”, “Notoginsenoside R1”. The literatures related to pharmacology and activity of Ast, Cal, For, DSS, PCA, RSA, Rg1, Rb1 and R1 were manually read. A DEG was considered as a potential target of a compound if it was found to be affected directly by the compound in a paper. For each paper, the information of target, PubMed ID, and CVD relevancy were recorded. Detailed information on the references used in this study can be found in **Table S1**.

### Experimental Target Validation

CVD-related DEGs were validated by manually checking published literature in PubMed (by April 10^th^, 2013). However, there were no previous studies reporting the targets of ENL, SDL and RDL. These three sesquiterpenes were isolated from volatile oils from Jiangxiang, which was reported to have anti-inflammatory activities, and was also used as a penetration enhancer [26]. Extracellular signal-regulated kinase-1/2 (ERK1/2), peroxisome proliferator-activated receptor-gamma (PPARγ) and heme oxygenase-1 (HO-1), which play important roles in inflammation, were identified as the potential targets of ENL and SDL. The anti-inflammatory activity of these targets was validated using LPS-stimulated RAW264.7 macrophage cells as models.

### Cell Viability Analysis by MTT Assay

Murine macrophage cell line RAW264.7 was maintained in DMEM medium supplemented with 10% heat-inactivated fetal bovine serum at 37°C in 5% CO_2_. To determine the effect of ENL and SDL on cell viability, RAW264.7 cells were seeded in 96-well plates at concentrations of 5000 cells/well. Following overnight incubation, cells were treated with several concentrations of ENL, RDL and SDL for 24 h. Cell viability was determined by the MTT assay. For MTT assay, the media were replaced with fresh DMEM medium that containing 10% FBS, penicillin/streptomycin, and MTT (0.5 mg/ml). After 4 h of incubation at 37°C, the media were removed, and replaced with 150 µL/well of DMSO. After mixing at 400 rpm in Thermomixer comfort (Eppendorf) at 37°C for 10 min, absorbance at 550 nm was measured on a Bio-Tek micro-plate reader. Each assay was conducted in triplicate and repeated three times.

### Nitric Oxide Assay

RAW 264.7 cells (4×10^4^ cells in 96-well microplate) were stimulated with 200 ng/ml LPS in 37°C CO_2_ incubator for 24 h. Nitric oxide (NO) production was detected by measuring nitrite levels in the culture media using the Griess reagent according to the manufacturer's instructions (Beyotime Institute of Biotechnology, China). Absorbance was measured at 550 nm.

### Effects of U0126, T0070907, and Pio on Nitric Oxide Secretion

U0126, a selective ERK1/2 phosphorylation inhibitor, was used to incubate with RAW264.7 macrophages together with LPS (200 ng/ml) or compounds with different concentrations plus LPS (200 ng/ml). PPARγ phosphorylation inhibitor T0070907 and PPARγ agonist Pio were also used in the same way as that of U0126. The concentration of U0126, T0070907 and Pio were used at 10 µM. NO production was determined using Griess Reagent. Each assay was conducted in triplicate and repeated three times, and outliers were excluded.

### Western Blot Analysis

For western blot assay, RAW264.7 macrophages were seeded into 60-mm dishes, cells were cultured in the 37°C CO_2_ incubator overnight. The second day, cells were treated with different compounds. The cells were collected after 24 h treatment. Cells were gently rinsed with ice-cold PBS. Then, 300 µL of cold RIPA buffer (RIPA lysis buffer, Cat No. P0013B, Beyotime Institute of Biotechnology, Haimen, Jiangsu, China) plus PMSF (Cat No. ST506, Beyotime Institute of Biotechnology, Haimen, Jiangsu, China), complete Mini (Cat No. 04693124001, Roche) and PhosSTOP (Cat No. 04906845001, Roche) were added to each dish. All steps of protein extraction were carried out on ice. Cells were lysed completely within 10 min, and the protein lysate was transferred to 1.5 ml centrifuge tubes, then the tubes were centrifuged at 12000 rpm for 10 min at 4°C. The supernatant in each tube was carefully collected without disturbing the sediments. Protein concentrations in the collected supernatants were determined using BCA protein assay kit (Cat No. P0012, Beyotime Institute of Biotechnology, Haimen, Jiangsu, China).

Protein samples containing LDS Sample Buffer (4×, NuPAGE, REF. NP0007) were heated at 70°C for 10 min and loaded onto a NuPAGE 10% Bis-Tris Gels, 1.5 mm, 15 well (Invitrogen, REF. NP0316BOX). PageRuler Prestained Protein Ladder (Fermentas, Cat No. SM0671) molecular weight marker used. The gels were run at 150 volts for 1 hour at room temperature (XCell SureLock Electrophoresis Cell). Proteins were transferred onto a PVDF membrane (iBlot, Gel Transfer Stacks, Mini, REF. IB401002) using an iBlot 7-minute Blotting System (Life technologies, Invitrogen). Primary antibodies for ERK1/2, pERK1/2, PPARγ and HO-1 were diluted in 5% BSA according to their manufacturer instructions respectively. GAPDH (Cat No. AG019, Beyotime) was used as the internal reference. Then the primary antibodies were incubated overnight at 4°C with 5% BSA. HRP-linked anti-rabbit IgG secondary antibody (Cat No. A0208, Beyotime) was used at 1∶1000 for ERK1/2, pERK1/2 and PPARγ, while HRP-linked anti-mouse IgG secondary antibody (Cat No. A0216, Beyotime) was used at 1∶1000 for HO-1. Detection was performed using SuperSignal West Femto Maximun Sensitivity Substrate (Cat No. 34095, Thermo Scientfic) on a BIO-RAD ChemiDoc XRS System.

### Network Construction and Network Analysis

The DEGs related to CVD and the main compounds were used as input in ArrayTrack v.3.5.0 to carry out pathway enrichment analysis, in which the MI or CVD associated pathways were selected for further analysis. Then, we constructed compound-target-pathway network and compound-pathway network in Cytoscape [16]. A compound and a target were connected with a solid line if the link was reported in at least one paper (≥1 literature) when they were; and the edge between target and pathway was constructed using the relationship found in ArrayTrack v.3.5.0 pathway enrichment analysis [27].

### Statistical Analysis

All experiments were performed three times in triplicate. Results are expressed as mean ± SD. Two-tailed unpaired Student's *t* tests were used to compare two distinct groups. When more than three groups were compared, One-Way ANOVA was performed between different treatment groups using PASW Statistics 18 (SPSS Inc., USA). A *p* value <0.05 was considered to be statistically significant.

## Results

### DEGs Analysis

312 genes (442 probe sets) in CHD@ZJU V1.0 database were found to be differentially expressed, out of which 83 genes with reverse rate (RR)>0.5 and fold change (FC)>1.1 after the QSYQ treatment were selected for further analysis (**Table S2**). Combined with the targets information about the compounds collected manually from published papers, 52 genes were included in this study, with additional information about compound, target, pathway, category and Fisher P-value (**Table S3**). The number of targets and the pathways of the compounds are listed in **Table 1**.

**Table 1:** The number of targets and pathways of 9 main compounds from QSYQ.

	Potential targets			
	Total	Confirmed in CVD-related references	Confirmed in non-CVD-related references	Pathways
Astragaloside IV	16	7	9	16
Calycosin	10	8	2	14
Formononetin	13	1	12	14
Danshensu	7	0	7	13
Protocatechuic Aldehyde	5	1	4	12
Rosmarinic Acid	21	0	21	15
Ginsenoside Rg1	26	7	19	16
Ginsenoside Rb1	21	4	17	16
Notoginsenoside R1	5	3	2	9

The numbers listed in the column of Pathway stands for CVD related pathways enriched in ArrayTrack v.3.5.0 pathway enrichment analysis of the targets of certain compound.

### Effect of ENL, RDL and SDL on RAW264.7 Macrophages Viability

After 24 h co-incubation with different concentrations (50, 20, 10, 5 µM) of ENL, RDL and SDL, MTT assay results showed that there was no obvious cytotoxicity of the three compounds (**Fig. 1B**) at these concentrations. Concentrations ≤50 µM were used for subsequent experiments.

### Effects of ENL and SDL on NO Production in LPS-stimulated RAW264.7 Macrophages

Two sesquiterpene compounds (ENL and SDL) from the volatile oil of *Dalbergia odorifera* T. Chen were able to suppress NO secretion from LPS-induced RAW264.7 macrophages. Both ENL and SDL significantly inhibited the NO secretion by LPS-stimulated RAW264.7 cells at 50 µM (*p*<0.01 for ENL, *p*<0.05 for SDL compared to model) (**Fig. 1C**). However, RDL at all concentrations had no obvious inhibition effect on NO secretion by LPS-stimulated RAW264.7 cells. Indomethacin at 25 µM was used as the positive control. More details of indomethacin, NEL and SDL on NO production can be found in **Fig. 1C**.

### Effects of ENL, RDL and SDL on ERK1/2, pERK1/2, PPARγ and HO-1 Expression

**Figure 2:**
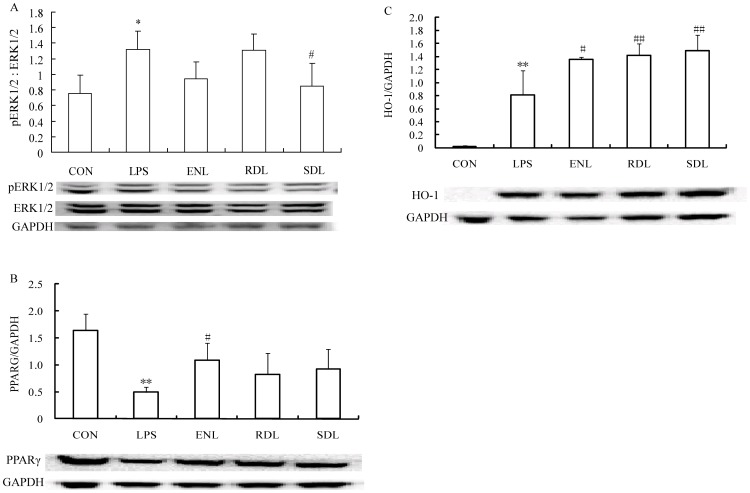
Effects of three sesquiterpene compounds from volatile oil of *Dalbergia odorifera* T. Chen on pERK1/2, ERK1/2, PPARγ and HO-1 expression in LPS-stimulated RAW264.7 macrophages. (A). LPS co-incubation induced significant increase of pERK1/2 expression, the ratio of pERK1/2 to ERK1/2 was higher than that in the control group (*p<0.05). 50 µM ENL attenuated the increased ratio of pERK1/2 to ERK1/2, while 50 µM SDL suppressed this ratio compared to LPS treated alone (#*p*<0.05), and RDL had no effect on the increased ratio. (B). LPS decreased PPARγ expression (**p<0.01 vs. control), and ENL, RDL, SDL all partially increased PPARγ expression, ENL showed the most strong effect (#*p*<0.05 vs. model). (C). The expression of HO-1 was induced to increase with 200 ng/ml LPS stimulation (**p<0.01 vs. control), and ENL, RDL, SDL further promoted HO-1 expression (for ENL *p*<0.05, and for RDL, SDL *p*<0.01, comparing with model). The RAW264.7 was stimulated with 200 ng/ml LPS for 24 h, and the concentration of ENL, RDL and SDL were all at 50 µM. Each assay was conducted in triplicate and repeated three times. ENL is trans-nerolidol; RDL is (3S,6R,7R)-3,7,11-trimethyl-3,6-epoxy-1,10-dodecadien-7-ol. SDL is (3S, 6S, 7R)-3,7,11-trimethyl-3,6-epoxy-1,10-dodecadien-7-ol.

Western blot results (**Fig. 2**) showed that LPS treatment significantly increased pERK1/2 to ERK1/2 ratio (*p<0.05 vs. control) and HO-1 levels (**p<0.01 vs. control), but decreased PPARγ expression in RAW 264.7 macrophages (**p<0.01 vs. control). Both ENL and SDL attenuated the increased ratio of pERK1/2 to ERK1/2 at 50 µM compared to LPS treated alone (#*p*<0.05 for SDL), but RDL had no effect on the increased ratio (**Fig. 2A**). While ENL, RDL, SDL all partially increased PPARγ expression, ENL showed the strongest effect (#*p*<0.05 vs. model) (**Fig. 2B**). ENL, RDL, SDL further promoted HO-1 expression (for ENL *p*<0.05, and for RDL, SDL *p*<0.01, comparing with model) (**Fig. 2C**).

### Effects of U0126, T0070907, and Pio on Nitric Oxide Secretion

**Figure 3:**
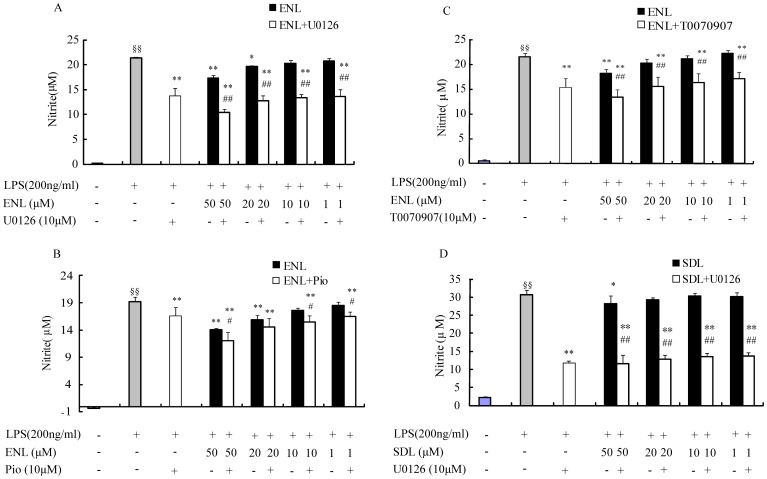
Effects of U0126, T0070907, and Pioglitazone hydrochloride (Pio) on NO production in LPS-stimulated RAW264.7 macrophages. LPS significantly induced NO secretion (^§§^ *p*<0.01 vs control). ERK1/2 phosphorylation antagonist U0126 significantly inhibited NO production (**p<0.01 vs. LPS treated alone) (A and D). PPARγ phosphorylation inhibitor T0070907 strongly suppressed NO release (**p<0.01 vs. model) (C), while PPARγ agonist Pio decreased NO secretion (**p<0.01 vs. model) (B). ENL does-dependently attenuated NO secretion, U0126, T0070907, and Pio further strengthened ENL's effect. 50 µM SDL also showed NO inhibition activity, while U0126 enhanced SDL's effect. * Means compared with model group (200 ng/ml LPS treated alone), and # stands for statistic significance analysis between two groups with and without small molecular tool drugs (*i.e.* U0126, T0070907, and Pio). ENL is trans-nerolidol; RDL is (3S,6R,7R)-3,7,11-trimethyl-3,6-epoxy-1,10-dodecadien-7-ol.

U0126, an antagonist of ERK1/2 phosphorylation, Pio, a PPARγ agonist, and T0070907, a PPARγ phosphorylation inhibitor, were able to inhibit NO secretion by LPS-induced RAW264.7 macrophages. So these three tool drugs were used to validate the effects of ENL and SDL. 200 ng/ml LPS significantly induced NO secretion (^§§^ *p*<0.01, vs control by student's t test) (**Fig. 3**). Combined-treatment with either of the three drugs, U0126, T0070907, and Pio, significantly strengthened the effect of ENL on suppressing NO secretion except 20 µM ENL plus Pio (**Fig. 3A, B, C**). U0126 also significantly enhanced the effect of SDL on inhibition of NO production (##*p*<0.01, vs. the group with SDL treated alone) (**Fig. 3D**).

### Network Construction and Pathway Analysis

**Figure 4:**
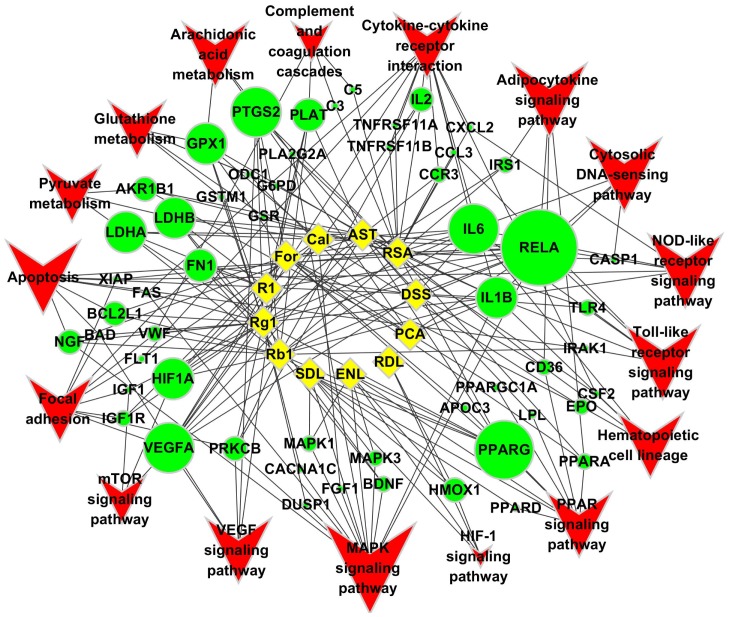
Multi-compound-multi-target-multi-pathway network of TCM QSYQ on treating myocardial infarction. There were 52 differentially expressed genes which were validated in published literatures. Node size of targets and pathways are proportional to the number of compounds associated with the node. This network depicted a clear three-level structure of the mode of action of main compounds of QSYQ, that is multi-compound regulated multi-pathways through modulating of groups of genes to show their pharmacological and clinical effects. Compound was linked to targets, while target were linked to pathways, a certain compound linked indirect link to pathways through certain targets. Diamond represents compounds; Circle represents target genes; Vee represents CVD related pathways. The edge went through compound to target, and target to pathway.

Combining the relationships in **Table S3** and the relationships from the experimental validation part, that is the potential targets such as PPARG (targets of ENL and SDL), HMOX1 (targets of ENL, RDL and SDL), MAPK3 and MAPK1 (targets of ENL and SDL) were also used to construct the network (**Fig. 4**). Then the paired relationships information (link between compound and target genes, link between target gene and pathways) was imported to Cytoscape software, and a compound-target-pathway network was constructed (**Fig. 4**). The three-level network consisted of 84 nodes (12 compounds, 55 genes and 17 pathways) and 230 edges. The detailed information of the compound names, gene names, pathway names and their category, and Fisher P-values can be found in **Table S3**. Through the visualization and network analysis of the relationships among compound, target gene and pathway in the constructed network, 52 DEGs were affected by at least one compound, in which 14 genes (*DUSP1, EPO, FGF1, FLT1, FN1, HIF1A, LDHA, LDHB, PLAT, PRKCB, RELA, VEGFA* and *VWF*) were the potential targets of the compounds based on the CVD-related literatures in PubMed. Among the 52 genes, RELA was influenced by 9 compounds. RELA is associated with cell growth and death, immune system, and endocrine system. IL6, PTGS2 and VEGFA were affected by 6 compounds, which maybe related with inflammation and angiogenesis. GPX1, HIF1A, IL1B, LDHA, LDHB and PPARG were affected by 5 compounds. FN1 and PLAT were influenced by 4 compounds. While five targets were influenced by 3 compounds, nine targets were influenced by 2 compounds, and the rest twenty six were affected by only one compound.

Using the 52 DEGs, 16 KEGG pathways were enriched in the ArrayTrack v.3.5.0 pathway enrichment analysis (**Table 2**), in which apoptosis signaling pathway is a critical pathway in regulating cell death. Most of the pathways, such as Cytokine-cytokine receptor interaction, NOD-like receptor signaling pathway, PPAR signaling pathway, Toll-like receptor signaling pathway, MAPK signaling pathway, Hematopoietic cell lineage, Cytosolic DNA-sensing pathway, Complement and coagulation cascades and Arachidonic acid metabolism, are either directly involved in immune and inflammation or play important roles there. In addition, there are several energy metabolism associated pathways including PPAR signaling pathway, Adipocytokine signaling pathway, Pyruvate metabolism. The Glutathione metabolism is antioxidant related, Focal adhesion plays role in cell motility and structure, VEGF signaling pathway has function in vasodilation, Complement and coagulation cascades is related with coagulation, and mTOR signaling pathway plays a role in hypoxia induced angiogenesis.

**Table 2:** 16(DEGs) affected by nine compounds from the TCM QSYQ.

Pathway	Category	Fisher P-value
Apoptosis	Cell Growth and Death/Cellular Processes	0.00001765
Cytokine-cytokine receptor interaction	Signaling Molecules and Interaction/Environmental Information Processing	0.0000376
NOD-like receptor signaling pathway	Immune System/Cellular Processes	0.00015053
PPAR signaling pathway	Endocrine System/Cellular Processes	0.00027289
Glutathione metabolism	Metabolism of Other Amino Acids/Metabolism	0.00047522
Focal adhesion	Cell Communication/Cellular Processes	0.00122324
Adipocytokine signaling pathway	Endocrine System/Cellular Processes	0.00182003
Toll-like receptor signaling pathway	Immune System/Cellular Processes	0.0020686
MAPK signaling pathway	Signal Transduction/Environmental Information Processing	0.00263886
VEGF signaling pathway	Signal Transduction/Environmental Information Processing	0.00317584
Hematopoietic cell lineage	Immune System/Cellular Processes	0.0059589
Cytosolic DNA-sensing pathway	Immune System/Cellular Processes	0.00622074
Complement and coagulation cascades	Immune System/Cellular Processes	0.01287548
Pyruvate metabolism	Carbohydrate Metabolism/Metabolism	0.01558565
mTOR signaling pathway	Signal Transduction/Environmental Information Processing	0.03115849
Arachidonic acid metabolism	Lipid Metabolism/Metabolism	0.04113971

## Discussion

Based on the results of microarray data and published literature information available in PubMed, a compound-target-pathway network (**Fig 4**) was constructed for the multi-compound-multi-target-multi-pathway mode of action (MOA) of the Chinese medicine QSYQ. The cardioprotective or anti-MI function of QSYQ is complex, involving modulation of groups of targets and a number of associated pathways. Cardiomyocytes apoptosis occurs promptly following acute myocardial infarction (AMI), and apoptosis pathway (including the mitochondrial death pathway) plays a pivotal role in this pathological process [28]–[30]. Inflammation accompanies from the initial stage of AMI and participates in the development of ischemia injury, one of the mechanisms is post-AMI hypoxia elicited inflammatory response [31].

Among the 16 pathways, nine are immune and inflammation related, including Cytokine-cytokine receptor interaction, NOD-like receptor signaling pathway [32], [33], PPAR signaling pathway [34]–[36], Toll-like receptor signaling pathway [37], [38], MAPK signaling pathway [39], Hematopoietic cell lineage, Cytosolic DNA-sensing pathway [40], Complement and coagulation cascades [41], [42] and Arachidonic acid metabolism [43]. Compounds from *Dalbergia odorifera* suppressed NO production in our in vitro target validation experiments. Anti-inflammation effect of QSYQ has been reported. QSYQ can reduce high sensitivity C reactive protein level in atherosclerotic rabbit to prevent the progress of atherosclerosis by inhibiting inflammatory reaction[44]. Also, it was reported that QSYQ can inhibit inflammatory effect through reducing TNF-α and IL-6 levels in rat after acute myocardial infarction[45], [46]. In clinical settings, QSYQ can improve cardiac function through anti-inflammatory effects by reducing TNF-α and IL-6 levels both in AMI and chronic heart failure patients[47], [48]. QSYQ could decrease the levels of high sensitivity C reactive protein, plasminogen activator inhibitor-1 and endothelin-1 in acute coronary syndrome patients[49].

Myocardial energy metabolism is another important event during and post-AMI, including fatty acid and glucose utilization. Acetyl-CoA is common end product of fatty acid, glucose and pyruvate to supply ATP. PPAR signaling pathway may prevent ischemic insult involving anti-inflammatory effect and promoting glucose utilization [36]. Adiponectin is a key adipocytokine having beneficial effect to facilitate fatty acid beta-oxidation and increase glucose utilization [50].

As for Glutathione metabolism pathway, glutathione has antioxidant activity [51] which is beneficial to prevent further myocardial injury elicited by reactive oxygen species (ROS) post-AMI. Focal adhesion plays a role in cell motility, cell proliferation, cell differentiation, and tissue remodeling after injury [52], [53]. VEGF signaling pathway has a function in vasculature vasodilatation through modulation of PKC, cPLA2, COX-2 and PGI2 [54]–[56]. Complement and coagulation cascades are activated early after injury. The modulation of coagulation may prevent secondary injury post AMI [41], [57]. mTOR signaling pathway plays a role in hypoxia-induced angiogenesis, which can help to recover blood supply to the ischemic myocardium post-MI [58], [59]. In addition, Hypoxia-Inducible Factor-1 signaling is also an important pathway which can be triggered by both hypoxia and inflammation conditions. The activation of Hypoxia-Inducible Factor-1 signaling could minimize the ischemic tissue damage and plays a protective role in inflammatory diseases[60].

The potential targets of those 9 main compounds from Huangqi, Danshen, Sanqi were validated using CVD-ralated literatures, and there was no reference reporting the targets of two main sesquiterpenes from Jiangxiang. In our experimental study, we investigated the anti-inflammatory effects of two main sesquiterpenes in volatile oils of Jiangxiang, i.e. ENL and SDL. SDL was newly identified from Jiangxiang in our previous study [21]. To the best of our knowledge, the pharmacological activities of ENL and SDL, and their potential protein targets have not been previously reported. We used nitric oxide assay, Western blot technology and small molecular compounds specific to the proteins of interest to validate the identified targets. As discussed above, ENL and SDL could suppress NO production in LPS-stimulated RAW264.7 macrophages.

Extracellular signal-regulated kinase-1/2 (ERK1/2) phosphorylation participates in stimuli induced inflammation. ERK1/2 phosphorylation was increased in lipopolysaccharide (LPS)-induced RAW264.7 macrophages. U0126 can suppress NO production in LPS-stimulated RAW264.7 macrophages by selectively inhibiting ERK1/2 phosphorylation, followed by inhibition of NF-κB transactivation and iNOS expression [61]. Inhibition of MAPKs (ERK1/2, p38MAPK, JNK) phosphorylation and NF-κB activation could decrease production of pro-inflammatory mediators (TNF-α, IL6, NO, *etc.*) [62]–[64]. In our study, U0126 suppressed NO production significantly (*p*<0.01 vs LPS treated alone) (**Fig. 3A, D**), and U0126 further reinforced NO inhibition induced by ENL (*p*<0.01 vs. ENL treated alone) and SDL (*p*<0.01 vs. SDL treated alone) (**Fig. 3A, D**). Therefore, ENL and SDL may attenuate LPS-induced NO release from RAW264.7 macrophages via inhibiting ERK1/2 phosphorylation; and NF-κB and iNOS may be involved in this process [65]. Furthermore, PPARγ plays an important role in inflammation [66], [67]. Activation of PPARγ leads to anti-inflammatory effects of PPARγ ligands (such as thiazolidinediones (TZDs), GW1929, indomethacin, ibuprofen and fenoprofen) [34], [35], [68]. LPS treatment has been shown to decrease PPARγ expression both in vivo and in vitro [69], the latter was repeated in our study (**Fig. 2B**). PPARγ ligand Pioglitazone inhibited LPS-stimulated microglia inflammation by inhibiting inflammatory mediators, including NO [70]. The effect of pioglitazone on NO synthase inhibition is potentially mediated through blocking of p38 MAPK activity [71]. Here in our study, 10 µM pioglitazone hydrochloride significantly inhibited NO production (*p*<0.01, **Fig. 3B**). ENL at 50 µM and 20 µM significantly suppressed NO secretion (*p*<0.01) (**Fig. 3B**). Combined use of Pio further enhanced the anti-NO production effect of ENL (**Fig. 3B**). Interestingly, phosphorylation of PPARγ decreases its transcriptional activity [72], [73]. T0070907 is the antagonist of PPARγ phosphorylation, it also inhibits ERK1/2 phosphorylation dose-dependently [74]. The results obtained in this study are consistent with those previously reported, as 10 µM T0070907 significantly inhibited NO release from LPS-stimulated RAW264.7 macrophages (*p*<0.01, **Fig. 3C**). 50 µM ENL attenuated NO secretion significantly (*p*<0.01, **Fig. 3C**), while 10 µM T0070907 further strengthened the effect of ENL (*p*<0.01, **Fig. 3C**). Therefore, ENL may inhibit NO production partially via inhibiting PPARγ phosphorylation and inducing PPARγ activity.

In cardiovascular disease, heme oxygenase-1 (HO-1) is another beneficial factor, it also plays an important role in anti-inflammation process [75]. LPS could enhance NO production through ROS generation, and induced HO-1 expression in RAW264.7 macrophages was able to inhibit iNOS expression [76]. Flavonoids could promote HO-1 expression, which suppresses LPS-induced NO production [77]. HO-1 was transcriptionally regulated by PPARγ [78], and PPARγ was able to stabilize HO-1 mRNA in monocytes/macrophages [79]. Carbon monoxide (CO) was proposed to be involved in the protective effects of HO-1, including its anti-inflammatory actions [80], [81]. LPS induced an increase in HO-1 expression in RAW264.7 macrophages, while ENL, RDL and SDL further enhanced HO-1 expression (*p*<0.05, *p*<0.01, and *p*<0.01, respectively, comparing with LPS treated alone, **Fig. 2C**).

We propose herein that the three sesquiterpene compounds (especially ENL and SDL) from volatile oil of Jiangxiang may suppress LPS-stimulated NO production from RAW264.7 macrophages at least partially via inhibition of ERK1/2 and PPARγ phosphorylation, and increasing expression of PPARγ and HO-1, in which NF-κB, ROS and iNOS maybe involved (**Fig. S1.**).

Chinese medicine treats diseases in a holistic way to make body recovery from imbalanced state by modulating a complex MOA including multiple pathways. Therefore, rodent animal models mimicking human disease are more suitable to evaluate the therapeutic effects of TCM and discover its MOA than cell culture. In our study, the underlying MOA of a clinical effective Chinese medicine QSYQ is systematically investigated combining mRNA expression profiles at molecular level on a rat myocardial infarction model. Using rat model is more convenient and easy to perform research to get rich information about the drug investigated both at phenotype and molecular base. On the other hand, cell culture model can be used to mimic a certain biological step of the drug investigated. Cell culture experiment has its merit as it is much easier to manipulate and to set investigation conditions. The results from animal experiments can be confirmed and studied in depth within cell culture experiments. However the results from animal studies should be carefully interpreted and concluded to explain the potential MOA of drug in treating human disease.

For the additional predicted targets as shown in **Fig. 4**, it is also important to validate them for a better understanding of the underlying MOA of QSYQ. Cell culture models play an important role in target validation. For example, inflammation related targets validation can be done with LPS-stimulated macrophage RAW264.7 cell culture model. TNF-α injured endothelial cell models are also used for anti-inflammatory related targets validation. The other two important processes mentioned in our study, apoptosis and energy metabolism can be studied with H9C2 cell line and primary neonatal rat heart cells to investigate cardioprotective effects and mechanisms of active compounds and drugs. Hypoxia or hypoxia-reoxygenation conditions are used to mimic in vivo ischemia or ischemia-reperfusion conditions. In addition, siRNA technology and target specific small molecular tool drugs could help to further confirm the validation results. Secondly, the targets validated at the in vitro experiments need further in vivo experiments confirmation. Rat MI model can be used to investigate the contributions of several targets to the anti-MI effects of compounds. The mRNA and protein expression profiles of specific targets after the intervention of single compounds or compounds combinations are tested using RT-PCR and western blot technologies. Finally, transgenetic mice models (both knock out and knock in) if available would provide more conclusive information for validating predicted targets with regard to cardioprotective effects.

The approach described in this paper provided an example on discovering the multi-compound, multi-target, multi-pathway MOA of a clinically effective Chinese medicine. A few steps have to be followed to ensure successful results. First, it is important to choose a clinically effective Chinese medicine with appropriate animal model available. Second, there should be reasonable understanding of the chemical compositions of the Chinese medicine. Third, in vitro experiments using cell culture models should be applied to test the pharmacological protective effects of potential compounds. Animal models (usually rodents) mimicking human disease are also used to confirm the effectiveness of potential compounds. Through these investigations, we understand the efficacious material basis of the Chinese medicine. Finally, network pharmacology analysis can be applied to integrate all these information to gain a full understanding of the MOA of the Chinese medicine.

In conclusion, the potential targets of 12 major compounds of QSYQ were identified by integrating gene expression data analysis and literature mining. These compounds may regulate multiple pathways through modulation of groups of genes to exert their anti-MI effects, involving biological processes of anti-apoptosis, anti-inflammation, antioxidant, anti-coagulation, energy utilization facilitation and angiogenesis promotion. Potential targets of 9 main compounds were validated by CVD-related references, while potential targets of three sesquiterpene compounds were further experimentally validated for their roles in mediation of inflammation.

## Supporting Information

Figure S1 Proposed molecular mechanisms of the anti-inflammatory effects of two sesquiterpene compounds from volatile oil of *Dalbergia odorifera* T. Chen. ENL is proposed as an inhibitor of ERK1/2 phosphorylation and PPARγ phosphorylation, an agonist of PPARγ, via which ENL could suppress NO production from LPS-stimulated RAW264.7 macrophages. SDL is also an antagonist of ERK1/2 phosphorylation, through which SDL may attenuate NO secretion. HO-1 may also participated in the regulation process of these two naturally occurring products in anti-inflammation. ENL is trans-nerolidol; SDL is (3S, 6S, 7R)-3,7,11-trimethyl-3,6-epoxy-1,10-dodecadien-7-ol.
(TIF)

Table S1 The target, PubMed ID, and CVD relevancy about the 9 main compounds of QSYQ.
(XLS)

Table S2 Differentially expressed genes(DEGs) slected from CHD@ZJU V1.0 database with fold change (FC) and reverse rate (RR) information after the QSYQ treatment.
(XLS)

Table S3 52 Differentially expressed genes(DEGs) with corresponding compounds,together with the information of pathways,categories and Fisher P-value.
(XLS)
