# Supplementary figures and images for: A Network Pharmacology Study of Chinese Medicine QiShenYiQi to Reveal Its Underlying Multi-Compound, Multi-Target, Multi-Pathway Mode of Action
Source: PLoS One. 2014 May 9;9(5):e95004. doi: 10.1371/journal.pone.0095004 (PMC4015902; doi:10.1371/journal.pone.0095004)

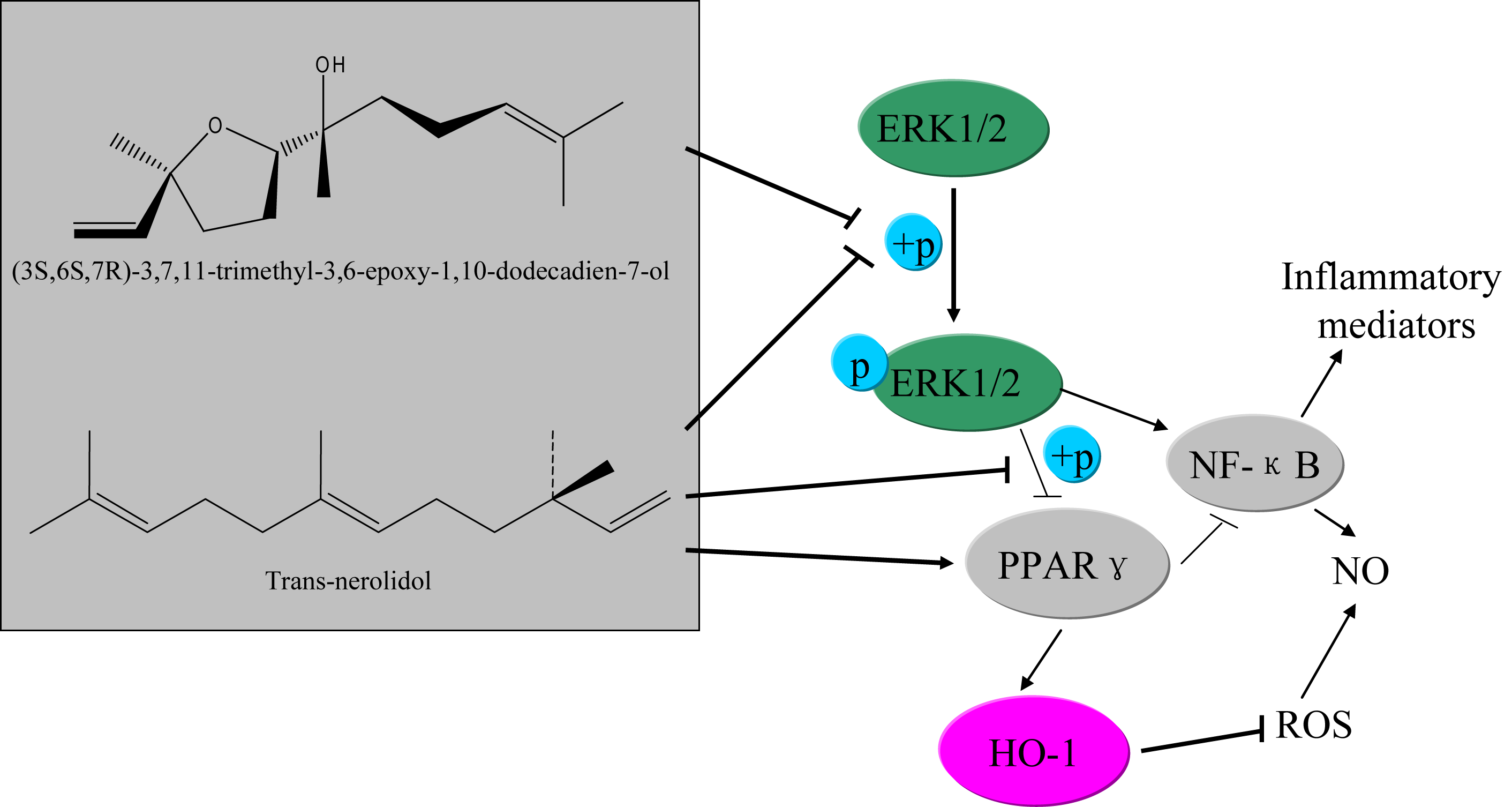

Supplement: Figure S1 — Proposed molecular mechanisms of the anti-inflammatory effects of two sesquiterpene compounds from volatile oil of Dalbergia odorifera T. Chen. ENL is proposed as an inhibitor of ERK1/2 phosphorylation and PPARγ phosphorylation, an agonist of PPARγ, via which ENL could suppress NO production from LPS-stimulated RAW264.7 macrophages. SDL is also an antagonist of ERK1/2 phosphorylation, through which SDL may attenuate NO secretion. HO-1 may also participated in the regulation process of these two naturally occurring products in anti-inflammation. ENL is trans-nerolidol; SDL is (3S, 6S, 7R)-3,7,11-trimethyl-3,6-epoxy-1,10-dodecadien-7-ol. (TIF) [file pone.0095004.s001.tif]
